# Necrotizing Fasciitis of the Forearm in a 20-Week Pregnant Woman: Case Report and Literature Review

**DOI:** 10.3390/diagnostics15040495

**Published:** 2025-02-18

**Authors:** Andreea Mironică, Bogdan Ioncioaia, Botond Janko, George Călin Dindelegan, Alexandru Ilie-Ene, Lucia-Ioana Furcovici, Balazs Sarkadi, Claudiu Ioan Filip

**Affiliations:** 1 Faculty of Medicine, University of Medicine “Iuliu Hatieganu” Cluj-Napoca, 1st Surgical Clinic, County Emergency Hospital, Street Clinicilor 3–5, 400347 Cluj-Napoca, Romania; ilieene.alexandru@gmail.com; 2 1st Surgical Clinic, County Emergency Hospital, Street Clinicilor 3–5, 400347 Cluj-Napoca, Romania; 3 Faculty of Medicine-6th Dept, 1st Surgery Clinic, “Iuliu Hațieganu” University of Medicine and Pharmacy, 400347 Cluj-Napoca, Romania; george.dindelegan@umfcluj.ro (G.C.D.); filip.claudiu.ioan@elearn.umfcluj.ro (C.I.F.)

**Keywords:** necrotizing fasciitis, pregnancy, forearm, skin graft

## Abstract

**Background and Clinical Significance:** Necrotizing fasciitis (NF) is a rare skin and soft tissue infection that progresses rapidly to necrosis and can be life-threatening. The incidence varies by geographic region but is generally low, with a mortality rate ranging between 11 and 22%. Early diagnosis and treatment are crucial for survival, particularly in patients with underlying conditions such as immune suppression, diabetes, obesity, trauma, recent surgical procedures, or renal pathology. However, the relationship between pregnancy and NF has not been extensively studied. **Case Presentation:** The case presented involves a 37-year-old, 20-week pregnant woman, who presented to the emergency department with septic shock and left forearm compartment syndrome. She reported no recent trauma or obvious source of contamination. The patient was immediately admitted and taken to the operating room. During admission, she underwent three surgeries, consisting of staged debridement, fasciectomy, and vacuum therapy and skin grafting. The patient was carefully monitored in the intensive care unit and multiple obstetrical consultations were performed to monitor the fetus. The patient was discharged with a fully integrated graft and with the donor area undergoing epithelialization. **Conclusions:** This case highlights the importance of early diagnosis and treatment of NF, particularly in high-risk patients, and the need for further research into the relationship between pregnancy and NF.

## 1. Introduction

The “necrotizing fasciitis” term was first used in 1952 by B. Wilson [1] to describe an infection of the fascial layers. The actual term defines a potentially life-threatening skin and soft tissue infection (incidence between 0.1 and 1 per 100,000 people per year [2]), rapidly progressive towards necrosis (mortality rates ranging from 20% to 30%, up to 73% [3]), requiring early diagnosis and urgent treatment. Necrotizing fasciitis (NF) is caused by various bacteria, with the most common being Group A Streptococcus (GAS), also known as *Streptococcus pyogenes*. The infection can be classified as either type I (polymicrobial) or type II (monomicrobial). Polymicrobial infections often involve both aerobic and anaerobic organisms, including *Staphylococcus aureus*, *Escherichia coli*, and *Clostridium* species. Monomicrobial cases are less frequent and typically involve Gram-positive bacteria [4]. Patients with concurrent diagnosis such as immune suppression, diabetes, obesity, trauma, or renal pathology have a higher risk of developing this disease. In relation to pregnancy, it is well known that it increases the risk of developing severe maternal infections (pneumonia, sepsis, malaria, hepatitis) [5], but the direct link between pregnancy and necrotizing fasciitis is yet to be documented [6].

## 2. Case Presentation

A 37-year-old 20-week pregnant woman presented to the ER in June 2022 with high intensity pain and edema of the left forearm and hand, which debuted 12 h before presentation. She had no history of recent upper limb trauma or an obvious contamination source. The pain level increased progressively with general malaise and hemodynamical instability. From the patient’s medical history, we mention upper respiratory infection 2 weeks before, C-section in 2021, and oxacillin allergy.

**Figure 1:**
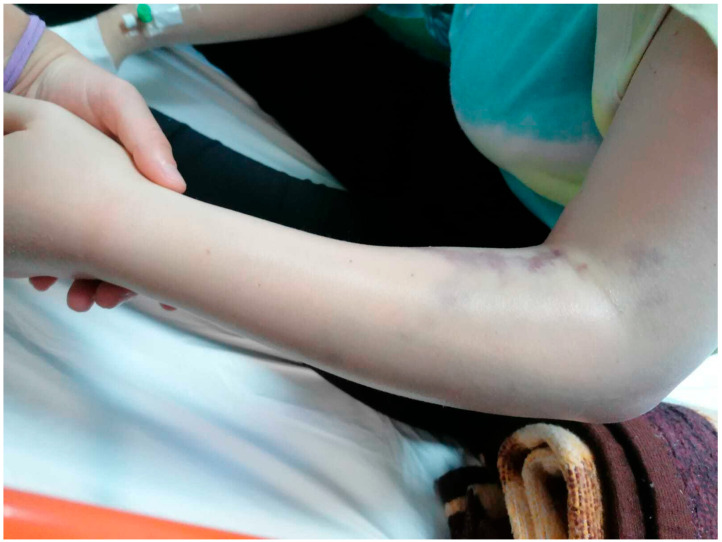
Swelling of the entire left forearm with multiple ecchymoses on the volar surface (in the ER department).

Local clinical examination on presentation revealed a warm left forearm and a hand with significant rash and edema, accompanied by increasing pain during both active and passive mobilization of the upper limb. The radial and ulnar pulses were imperceptible, capillary refill time (CRT) was >4 s, and multiple antebrachial ecchymoses were noted (Figure 1). The blood pressure (BP) was 130/80 mmHg, heart rate (HR) was 117 bpm, and the patient’s temperature was 36.8 °C.

The initial blood tests revealed leukocytosis with a white blood cell count of 12.91 (×10^9^/L), and elevated creatine kinase levels of 168 U/L. Additionally, other relevant values included C-reactive protein (CRP) at 31.94 mg/L, hemoglobin at 11.1 g/dL, sodium at 134 mmol/L, glucose at 118 mg/dL, and renal function markers with creatinine at 0.46 mg/dL.

The LRINEC (Laboratory Risk Indicator for Necrotizing Fasciitis) score at admission was 7. This score was calculated based on the following components:

C-reactive protein (CRP): 31.94 mg/L.

White blood cell count (WBC): 12.91 × 10^9^/L.

Hemoglobin: 11.1 g/dL.

Sodium: 134 mmol/L.

Creatinine: 0.46 μmol/L.

Glucose: 118 mg/dL.

Each of these components contributes to the overall LRINEC score, and their respective values provide insight into the risk level for necrotizing fasciitis. A score of 7 indicates a high risk for the condition, necessitating immediate and aggressive medical intervention.

Ultrasound examination showed no signs of venous thrombosis in the upper limb; suspicion of subcutaneous and intrafascial liquefaction along the affected area. Arterial flow was present up to the distal arteries.

The plastic surgery team established clinical forearm compartment syndrome. The patient was admitted immediately and taken to the OR—day 0.

**Figure 2:**
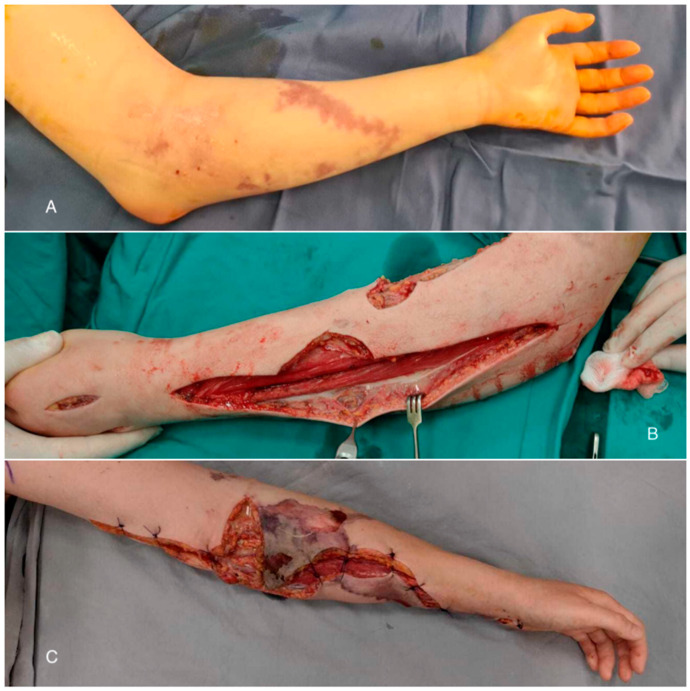
The gray fluid drainage after the decompression incisions ((**A**)—the forearm before the first surgery; (**B**)—the grey fluid externalized; (**C**)—the aspect of the forearm 24 h after the first surgery).

In general anesthesia, large decompression incisions were made from the carpal tunnel to the cubital fossa and on the dorsal aspect of the hand and forearm. During dissection, gray fluid externalized (Figure 2) was observed from the proximal third of the forearm serous. Fluid and fascia samples were collected for microbiological and histopathological examination, respectively. Decompression fasciotomies were performed on the volar aspect of the forearm along with fasciectomies.

After rigorous debridement of the macroscopically altered tissue and hemostasis, the forearm regained its normal color and a capillary pulse was present in each finger. Subsequently, abundant lavage with betadine, physiological serum and wound dressing with paraffin gauze, and sterile compresses were practiced. The patient was referred to the ICU for specific treatment and surveillance.

Because of the expansion of necrosis, further debridement was needed—on day 1—and 5 days from day 0. Excisions of the skin, subcutaneous tissue, and fascia from the forearm extending to the cubital fossa were practiced. Antebrachial neurovascular pedicles were explored and no pathological changes were observed. We started vacuum therapy at 80 mmHg pressure on day 5, achieving granulation tissue in 5 days. Subsequently, the defect was covered with autologous skin graft harvested from the left thigh—on day 10 from day 0.

During this period, the patient was carefully monitored in the ICU and received antibiotic treatment. A positive intraoperative secretion of *Streptococcus pyogenes* was detected, leading to an allergology consultation and the administration of penicillin G without incident (the initial empiric antibiotic therapy consisted of a combination of vancomycin and piperacillin–tazobactam). Additionally, the pregnancy was continuously monitored by obstetricians without any complications or risks to the mother or child.

**Figure 3:**
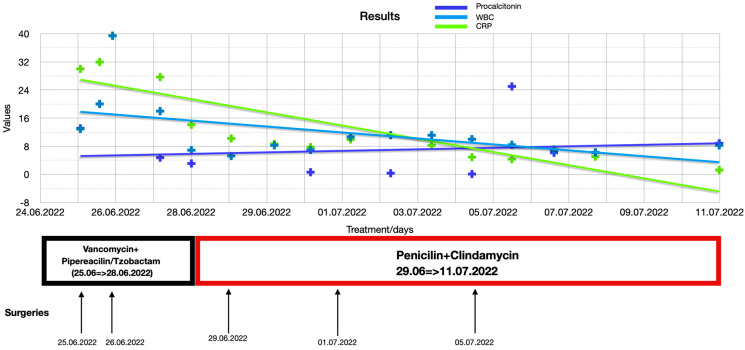
Inflammatory markers levels evolution during hospitalization.

The evolution of the patient in ICU was favorable, with normalization of biological samples and general condition, allowing the patient to transfer to the plastic surgery department on postoperative day 15—from day 0. The progression of inflammatory biomarkers (C-reactive protein, procalcitonin, and leukocytes) in relation to antibiotic administration and surgical interventions is depicted in Figure 3, and in Table 1 represents a table with the detailed values.

**Figure 4:**
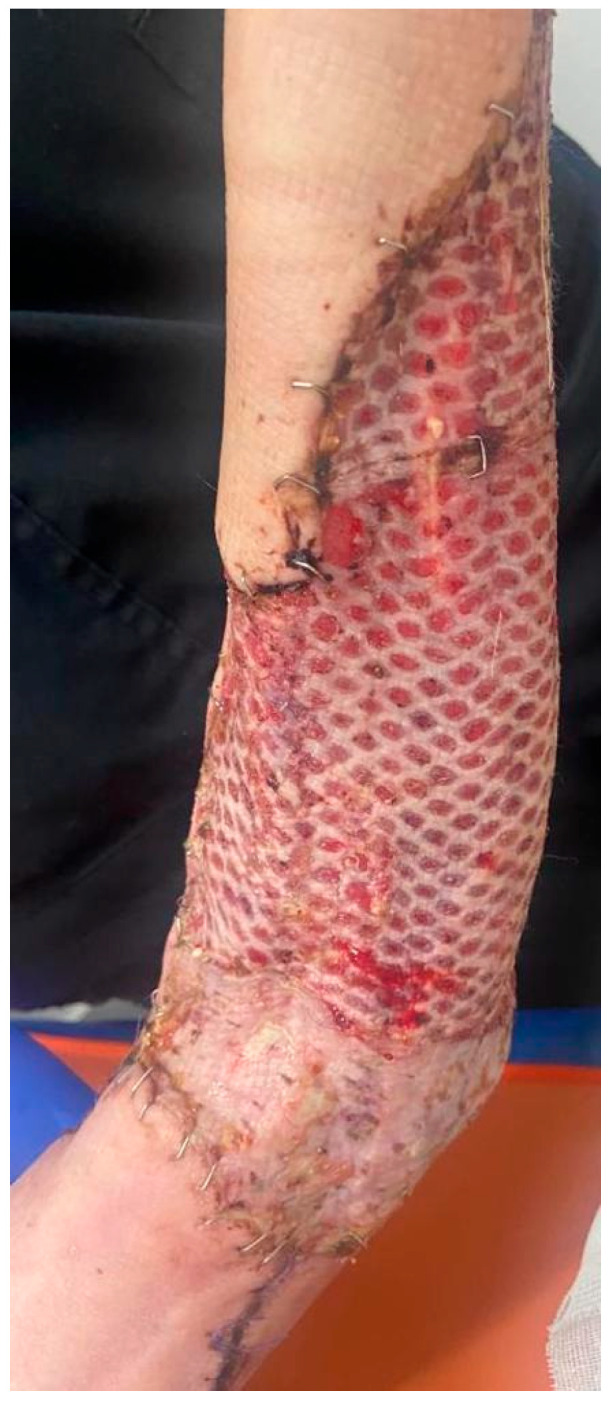
A week after discharge: fully integrated skin graft.

After three days (day 18 from day 0), the patient was discharged with fully integrated graft at the forearm level, with the donor area with undergoing epithelialization, and she was hemodynamically and respiratory stable, with normal blood samples (Figure 4).

The plastic surgery team regularly followed the patient until complete epithelialization in an outpatient regime.

**Figure 5:**
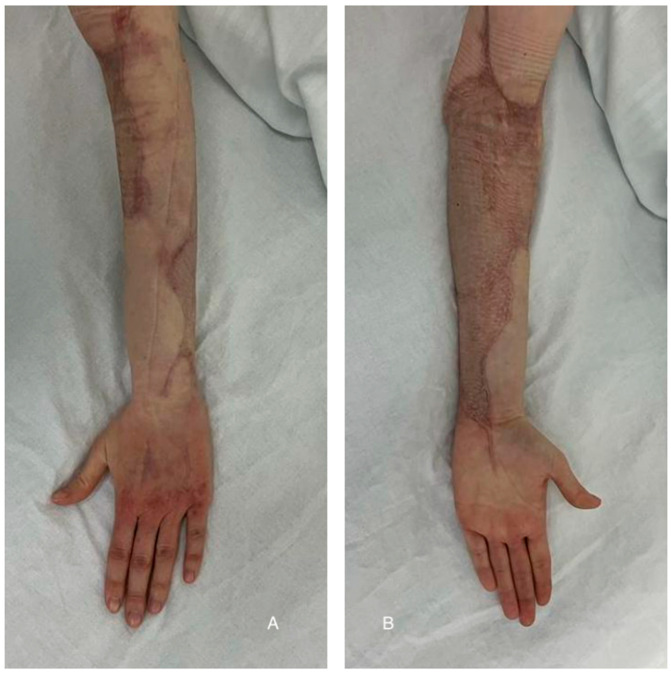
Eight months after surgeries: fully recovered functionality((**A**)—dorsal forearm and hand; (**B**)—ventral forearm and hand).

At 38 weeks, the patient spontaneously gave birth to a healthy baby girl without any other complications or incidents (Figure 5).

The patient in the postpartum period presented for a follow-up examination at 8 months post-surgery, with favorable local and general progress, scars under local treatment with silicone, and without functional limitations.

For a proper management of this complex case, a multi-modal team of doctors specialized in plastic surgery, gynecology, ICU, laboratory medicine, allergology, and pathological anatomy were involved.

## 3. Discussion

We present and publish this clinical case for teaching purposes: an interesting and difficult case to manage. After research in specialized databases, we found only one similar case of necrotizing forearm fasciitis in a pregnant woman [7]. In the specialized literature, multiple other studies can be identified that primarily focus on necrotizing fasciitis in pregnant women; however, in the majority of cases, the patients were in the postpartum period, following the termination of a pregnancy or other genital area interventions, making the point of entry easily identifiable [8,9,10]. Additionally, the preferred areas for this pathology in pregnant women or puerperal women are the abdomen, perianal area, or lower extremities [11,12].

Early diagnosis and treatment are essential. Compartment syndrome and necrotizing fasciitis more commonly occur in polymicrobial forms (type I) [13], and in this particular case monomicrobial infection with *Streptococcus pyogenes* (type II) was confirmed.

Group A Streptococcus (*Streptococcus pyogenes*) (GAS) is a rare but potentially fatal infection. GAS is the most common cause of necrotizing fasciitis, accounting for approximately 70–80% of cases. Other bacterial agents that can cause this condition include Staphylococcus aureus, Clostridium species, and other streptococcal species, but GAS is by far the most prevalent [14]. Even when antibiotics cure the most severe infections, the overall case fatality rate (CFR) for invasive infections caused by S. pyogenes is 15% to 20%, and if septic shock develops, the mortality rate is 40% to 60%. For obstetricians, GAS has a unique place in medical history because it is associated with puerperal fever in the mid-19th century, when up to 15% of all women giving birth in centralized hospitals died from this disease. Despite advancements in infection control protocols, Group A Streptococcus (GAS) infection continues to be a significant contributor to maternal morbidity and mortality during pregnancy and the puerperal period [15].

The need for staged surgeries are proof of the progressive character of this infection and are not unusual. Extended carcinological excisions (up to the level of the macroscopic undamaged tissues) were needed to control and limit the infection, thus respecting the protocol of treatment of this pathology.

The evolution of this pathology in patients with concomitant diseases that produce immunosuppression is described. In this case, pregnancy was the main and only cause of immunosuppression, and no history of local trauma was identified. A pregnant woman’s immune system undergoes several changes to allow for tolerance of the father’s fetal antigens. Cell-mediated downregulation of immunity occurs and maternal lymphocytes show reduced proliferative responses to soluble antigens and allogeneic lymphocytes [6,16].

Surgical treatment alone may not be sufficient [8], and selecting appropriate antibiotic therapy for the patient within a short time frame presents several challenges. In addition to considerations related to pathogen sensitivity and resistance [17], it was crucial to evaluate the potential risks to the pregnancy, including teratogenicity, fetal growth restriction, miscarriage, and preterm labor. Special attention was given to the potential for delayed fetal development and the possibility of preterm delivery, which can occur due to systemic infection and the aggressive treatments required for necrotizing fasciitis.

Moreover, we carefully assessed the patient’s potential drug allergies and tailored the treatment to minimize risks to both the mother and fetus. The antibiotics chosen were selected to balance the efficacy against the identified pathogen with the safety of the pregnancy [18]. Close monitoring of fetal well-being was maintained throughout the treatment course, and the pregnancy remained unaffected by the infection or the interventions required for treatment.

However, the risk of pregnancy complications such as preterm labor or fetal distress remains a significant consideration in cases of necrotizing fasciitis in pregnant patients, necessitating a multidisciplinary approach to manage both the maternal infection and fetal health effectively.

Penicillin is considered a first line treatment for *Streptococcus pyogenes* group A of beta-hemolytic streptococcus [19]. Consequently, in the case presented, the antibiogram confirmed sensitivity to penicillin. Taking into consideration a negative allergy test and the fact that penicillin can be safely used in pregnant patients, the treatment was initiated.

## 4. Conclusions

In summary, the successful management of the 37-year-old pregnant woman with forearm compartment syndrome highlights the effectiveness of a collaborative, multidisciplinary medical team. Swift diagnosis, urgent surgical intervention, and meticulous postoperative care, involving specialists in plastic surgery, gynecology, ICU, laboratory medicine, allergology, and pathological anatomy, led to a favorable outcome. The patient, who gave birth to a healthy baby girl, experienced a smooth postoperative recovery with no functional limitations, emphasizing the importance of a comprehensive and coordinated approach in complex medical cases.

## Figures and Tables

**Table 1:** Detailed values of inflammatory markers.

Days	WBC (× 10^9^/L)	CRP (mg/dL)	Procalcitonin (ng/mL)
25/06/2022 05:53	12.91	30.00	8.835
25/06/2022 17:526	20.02	31.94	
26/06/2022 01:12	39.42	39.42	6.151
27/06/2022 05:44	17.98	27.68	25.009
28/06/2022 00:29	6.87	14.11	
29/06/2022 00:23	5.23	10.22	13.090
30/06/2022 02:16	8.26	8.68	
30/06/2022 23:59	6.94	7.75	4.752
02/07/2022 00:01	10.66	9.92	3.082
03/07/2022 00:04	11.16	11.16	
04/07/2022 00:39	11.13	8.24	
05/07/2022 00:51	10.00	4.90	0.614
06/07/2022 00:48	8.40	4.33	
07/07/2022 02:05	6.90	7.18	0.320
08/07/2022 02:54	6.24	5.04	
11/07/2022 05:14	8.24	1.23	0.092

## Data Availability

No new data were created or analyzed in this study.
